# Supplementary material for: Leveraging uncertainty information from deep neural networks for disease detection
Source: Sci Rep. 2017 Dec 19;7:17816. doi: 10.1038/s41598-017-17876-z (PMC5736701; doi:10.1038/s41598-017-17876-z)
Supplement: Supplementary file 1 — Supplementary material [file 41598_2017_17876_MOESM1_ESM.pdf]

Leveraging uncertainty information from deep neural networks for disease detection: supplementary material

Christian Leibig[1,*], Vaneeda Allken[1], Murat Seçkin Ayhan[1], Philipp Berens[1,2+], and Siegfried Wahl[1,3+]

[1]Institute for Ophthalmic Research, Eberhard Karls University, Tübingen, Germany
[2]Bernstein Center for Computational Neuroscience and Centre for Integrative Neuroscience, Eberhard Karls University, Tübingen, Germany
[3]Carl Zeiss Vision International GmbH, Germany
[*]christian.leibig@uni-tuebingen.de
[+]Co-senior author

Performance improvement under decision referral for an already trained network

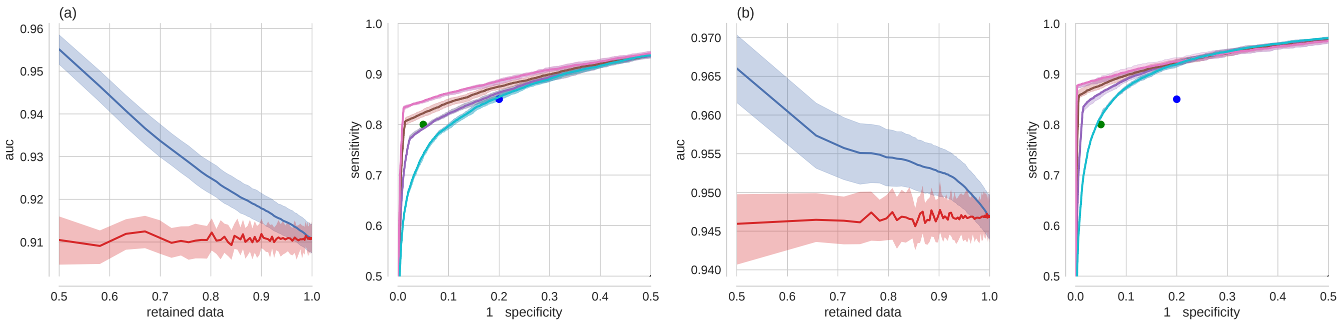

Figure S1. Application of uncertainty-informed decision referral to an existing network (a, left) ROC AUC over the fraction of retained data under uncertainty informed (blue) and random (red) decision referral for the JFnet, recast to detect disease onset 1. **(a, right)** ROC curves for all data (no referral: turquoise) and different fractions of retained data (90%: purple, 80%: brown, 70%: pink). National UK standards for the detection of sight-threatening diabetic retinopathy (in[2] defined as moderate DR) from the BDA (80%/95% sensitivity/specificity, green dot) and the NHS (85%/80% sensitivity/specificity, blue dot) are given in all subpanels with ROC curves. **(b)** same as (a), but for disease onset 2. All subfigures are based on Kaggle DR test images.

Relation between μ_{pred} and σ_{pred}

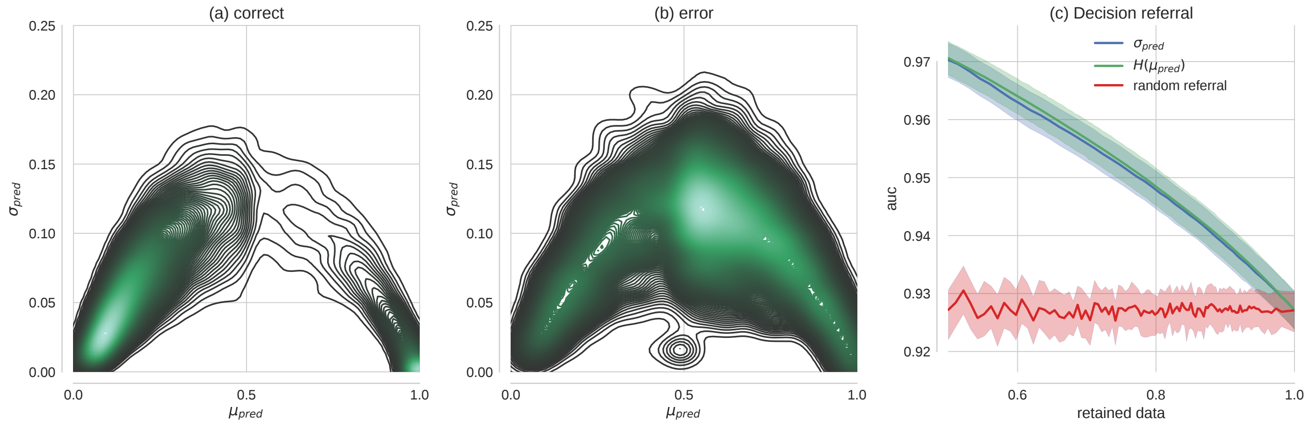

Figure S2. (a) & (b) Relation between first (μ_{pred}) and second (σ_{pred}) moments of approximate predictive posterior for correct (a) and erroneous (b) detection of moderate DR on Kaggle test images. μ_{pred} obtained via MC dropout is more related with σ_{pred} than the network output $p(diseased|image)$ obtained with standard dropout (compare fig. 2). **(c)** For comparison with our performance results obtained with σ_{pred} as uncertainty (fig. 4, S1), we quantified the uncertainty in terms of the binary entropy $H(p) = -(p \log p + (1-p) \log(1-p))$ as an alternative uncertainty measure which is applicable to both the Bayesian and conventional network output. When using $H(\mu_{pred})$ (green curve) we were able to achieve similar performance improvements under decision referral compared to when using σ_{pred} (blue curve) instead. Random referral is shown in red.

Gaussian processes

Gaussian processes (GPs) enable probabilistic kernel machines for solving regression and classification problems. The GP inference takes place in a function space and a kernel is a covariance function $k(\mathbf{x}^i, \mathbf{x}^j)$ that estimates the covariance of two latent variables $f(\mathbf{x}^i)$ and $f(\mathbf{x}^j)$ in terms of input vectors[3]. Given a *GP-prior*, e.g., $\mathbf{f} \sim \mathcal{N}(\mathbf{0}, \mathbf{K})$, over a set of latent variables, where $\mathbf{K}$ is a covariance matrix determined by the choice of covariance function $k(\cdot,\cdot)$, a *GP-posterior* $p(\mathbf{f}|\mathbf{X}, \mathbf{y})$, where $\mathbf{y}$ is the target vector and $\mathbf{X}$ is the data matrix, can be inferred in the light of the observed data, namely the likelihood $p(\mathbf{y}|\mathbf{f})$. In the process, the hyperparameters of the covariance function are also automatically tuned via likelihood maximization[3].

GP classification is essentially *binary* and class labels are in $\{-1,+1\}$. Similar to the case of the *logistic regression*, the goal is to assign $p(y^* = +1|\mathbf{x}^*)$ for an unseen test example $\mathbf{x}^*$. But, the underlying Bayesian treatment of GPs results in an *averaged predictive probability*[3], instead of a single point estimate, for $\mathbf{x}^*$ via Eqs.1 and 2.

$$\bar{\lambda}(f^*) = \int \lambda(f^*) p(f^*|\mathbf{X},\mathbf{y},\mathbf{x}^*) df^*, \text{ where} \tag{1}$$

$$p(f^*|\mathbf{X},\mathbf{y},\mathbf{x}^*) = \int p(f^*|\mathbf{X},\mathbf{x}^*,\mathbf{f}) p(\mathbf{f}|\mathbf{X},\mathbf{y}) d\mathbf{f}, \tag{2}$$

$\lambda(f^*)$ is a squashing function that maps its inputs into $[0,1]$. A common choice of $\lambda(f^*)$ is in Eq. 3.

$$p(y^* = +1|\mathbf{x}^*) = \lambda(f^*) = \frac{1}{1+\exp(-f^*)}. \tag{3}$$

The exact computation of the averaged predictive probability in Eq.2 is intractable and approximation methods are used to this end. A comprehensive review[4] of approximate inference methods for GP classification is available in literature. Two commonly used methods are Laplace Approximation (LA)[3] and Expectation Propagation (EP)[6].

LA replaces $p(\mathbf{f}|\mathbf{X},\mathbf{y})$ with a Gaussian approximation centered at the mode of $p(\mathbf{f}|\mathbf{X},\mathbf{y})$[3]. LA is a local method in the sense that it exploits the properties of the posterior at a particular location only, e.g., at the posterior distribution's mode[4]. Due to its simplicity, LA is very fast; however, it may lead to substantial underestimations of the mean and covariance, especially, in high-dimensional spaces because the mode and mean can be far from each other[4,5].

The EP is a general approximation tool that can perform approximate inference more accurately than other methods like Monte Carlo, LA and variational Bayes[6]. Thus, it is heavily used for GP learning. EP is a global method in the sense that it utilizes many local approximations in order to approximate the posterior[3,4]. The use of many local approximations results in a small global divergence between the posterior and its proxy[4]. As a result, EP delivers accurate marginals, reliable class probabilities and faithful model selection[4]. On the downside, the convergence of EP is not generally guaranteed and its runtime is 10 times longer in comparison with LA[4].

In summary, a GP classification can be made by just checking the sign of the predictive function sampled from Eq. 2. In this regard, if one only cares about the error rate or the computational resources are limited, LA may be a practical solution[4]. On the other hand, EP offers higher quality approximations and it should be considered first, despite the associated computational costs and risk of not converging, when the quality of approximation matters. In the face of practical challenges, such as non-convergence or exceptionally long runtimes, one can always fall back onto LA.

Neural Network Covariance Function

A shallow neural network with infinitely many hidden units and Gaussian weights, such as $\mathbf{w} \sim \mathcal{N}(\mathbf{0},\Sigma)$, converges into a GP that can be constructed with the following covariance function[3,7,8]:

$$k(\mathbf{x},\mathbf{z}) = \frac{2}{\pi} \sin^{-1}\left(\frac{2\tilde{\mathbf{x}}^T\Sigma\tilde{\mathbf{z}}}{\sqrt{(1+2\tilde{\mathbf{x}}^T\Sigma\tilde{\mathbf{z}})(1+2\tilde{\mathbf{x}}^T\Sigma\tilde{\mathbf{z}})}}\right), \tag{4}$$

where $\tilde{\mathbf{x}} = (1,\mathbf{x})^T$ is an augmented input vector and Σ is a weight covariance matrix. In other words, a GP with the neural network covariance function (Eq. (4)) emulates a shallow network.

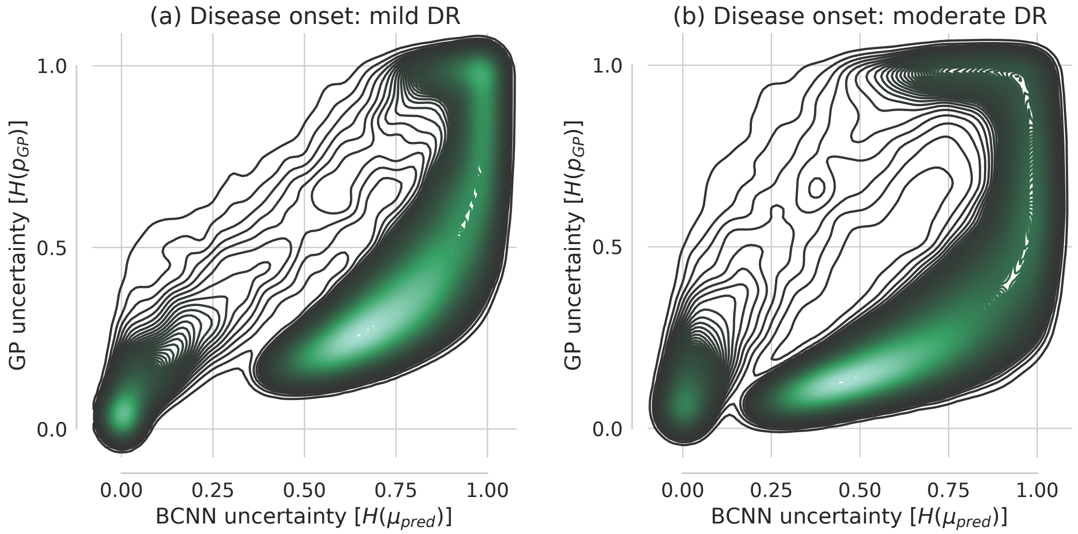

Figure S3. Comparison of Bayesian network (MC dropout) vs. Gaussian process (GP) uncertainty. (a) & (b) Density plots for uncertainties [entropy] obtained from BCNNs vs. GPs for detecting mild (a) and (b) moderate DR from Kaggle data respectively. For the majority of the data, the BCNN uncertainties tend to be larger than the uncertainties from the GP predictions.

References

1. Gal, Y. *Uncertainty in Deep Learning*. Ph.D. thesis, University of Cambridge (2016).
2. Younis, N., Broadbent, D. M., Harding, S. P. & Vora, J. P. Incidence of sight-threatening retinopathy in Type 1 diabetes in a systematic screening programme. *Diabetic medicine : a journal of the British Diabetic Association* **20**, 758–765 (2003).
3. Rasmussen, Carl Edward, Gaussian Processes for Machine Learning (Adaptive Computation and Machine Learning) 2005, The MIT Press, 026218253X.
4. Nickisch, Hannes, Rasmussen, Carl Edward, Approximations for binary Gaussian process classification, *Journal of Machine Learning Research*, **9**, 2035–2078, (2008).
5. Kuss, Malte, Rasmussen, Carl Edward, Assessing approximate inference for binary Gaussian process classification, *Journal of Machine Learning Research*, **6**, 1679–1704, (2005).
6. Minka, Thomas P. *A family of algorithms for approximate Bayesian inference*, Ph.D. thesis, Massachusetts Institute of Technology (2001).
7. Neal, Radford M., *Bayesian Learning for Neural Networks*, Springer-Verlag New York, Inc., Secaucus, NJ, USA, 0387947248, (1996)
8. Williams, Christopher K. I. and Barber, David, Bayesian Classification With Gaussian Processes, *IEEE Trans. Pattern Anal. Mach. Intell.*, **20**, 1342–1351, (1998).
